# Mapping the evidence on interventions to raise awareness on lung cancer in resource poor settings: a scoping review protocol

**DOI:** 10.1186/s13643-019-1138-x

**Published:** 2019-08-24

**Authors:** Siyabonga B. Dlamini, Benn Sartorius, Themba Ginindza

**Affiliations:** 1 School of Nursing and Public Health, Discipline of Public Health Medicine, University of KwaZulu-Natal, Durban, South Africa; 2 Health Metrics Sciences, School of Medicine, University of Washington, Seattle, USA; 3 Faculty of Infectious and Tropical Diseases, Department of Disease Control, London School of Hygiene and Tropical Medicine, London, UK

**Keywords:** Lung cancer, Community awareness, Interventions, Resource limited settings and effective

## Abstract

**Background:**

Lung cancer is the most commonly diagnosed cancer with 11.6% of the total cases attributable to lung cancer. It is currently the leading cause of death among cancer-related deaths worldwide. This is a major public health concern. Death due to lung cancer is preventable with interventions encouraging early presentation, diagnosis, smoking cessation and prompt and proper treatment. Literature shows that people are willing to screen for lung cancer if they understand the related risk, because of their behaviour, thus, highlighting the need for tailored interventions to address the associated risks. The aim of the review is to map the available literature on interventions raising community awareness about lung cancer (knowledge, attitudes and health-seeking behaviour) and effectiveness thereof among adults in resource-poor settings.

**Methods and analysis:**

A methodological framework of Arksey and O’Malley will be used to guide this scoping review of published data. This process will start by searching several databases, including the Cochrane Library, PubMed, Scopus, MEDLINE, the Cumulative Index to Nursing and Allied Health Literature (CINAHL), PsycInfo, Web of Science, Google Scholar and the Educational Resources Information Centre (ERIC). A two-stage process will be done, where, firstly, two reviewers will independently screen the titles and abstracts for eligibility to be included in the final selection of studies. Secondly, a full-text screening of the articles from selected titles and abstracts will then be conducted. A tool developed through an iterative process by the researchers will be used to analyse all bibliographic data and study characteristics of selected studies.

**Discussion:**

The results will be used to inform policy and practice in terms of developing interventions on lung cancer awareness. The results of this scoping review will be disseminated through scientific publication, conferences and future workshops with health care professionals involved in lung cancer awareness campaigns.

**Electronic supplementary material:**

The online version of this article (10.1186/s13643-019-1138-x) contains supplementary material, which is available to authorized users.

## Background

Cancer morbidity and mortality is increasingly becoming a major public health problem, and it is the second leading cause of death worldwide [1]. Globally, over 20 million new cancer cases are projected for 2025 compared to about 14.1 million and 17.5 million new cases in 2012 and 2015, respectively [1–4]. Global cancer cases increased by 33% between 2005 and 2015 [1]. Lung cancer accounted for about 1.8 million of all cancer cases diagnosed in 2012 [4]. Lung cancer is the most commonly diagnosed cancer with 11.6% of the total cases attributable to lung cancer [5]. There were over 1.5 million deaths in 2012 due to lung cancer [4]. Lung cancer is currently the leading cause of death among cancer-related deaths worldwide [5, 6] and is thus a major public health concern. Low- and middle-income countries (LMICs) face an increasing challenge of cigarette use and availability of tobacco products [7–9]. Death due to lung cancer is preventable with interventions encouraging early presentation and diagnosis, smoking cessation and prompt and proper treatment [6, 9–11]. Islami and colleagues argued that prevention interventions were needed to curb lung cancer morbidity and mortality in most African countries and other LMICs as a result of increased use of tobacco products [9].

A study conducted in Malaysia among smokers and non-smokers showed that people were willing to go for screening, if they were informed that they were particularly at high risk of lung cancer, especially if they were smokers [11]. Whilst prevention is undeniably the best way to address lung and other cancers, challenges brought about by modern lifestyles are not making this approach any easier. For example, modern lifestyles, socio-economic developments and technologically complex human and built environments have profound effects on the scale and profile of cancer; hence, LMICs are now experiencing the greatest increase in cancer incidence [3].

Williams et al. [12] propose multiple interventions including cancer awareness, advocacy, research, workforce care training and funding to avert this situation. Furthermore, there is poor access to quality of care for cancer in SSA, and South Africa is less than optimal [13]. An overwhelming majority of lung cancer patients are diagnosed late and are thus initiated on treatment late. This reduces the effectiveness of treatment, which results in the majority of lung cancer patients dying whilst on treatment [14–17]. This suggests that there are barriers to early diagnosis of lung cancer. These barriers may include (i) structural factors, where patient pathways of care may hinder early diagnosis, (ii) lack of proper health-seeking behaviour, where the patients do not go to health facilities early so that they are screened and initiated on life-saving treatment early, and (iii) cultural beliefs that serve as barriers for individuals to seek help early [16, 18–20]. Hence, there is a need for better awareness and early detection.

This article outlines the protocol for a scoping review of published literature focusing on interventions that have been proposed and/or implemented to raise community awareness about lung cancer (knowledge, attitudes and health-seeking behaviour) among adults in resource-poor settings.

## Methods

### Protocol design

The scoping review is informed by the framework proposed by Arksey and O’Malley [21] which has been further developed by Levac et al. [22] and the Joanna Briggs Institute [23]. This framework recommends to organise the review process in at least five stages [21]. These stages are (1) identifying the research question, (2) identifying relevant studies, (3) study selection, (4) charting the data, and (5) collating, summarising and reporting the results.

These stages and how they will be applied in the proposed scoping review are discussed below.

### Framework stage 1: Identifying the research question

The design of this protocol has been informed by the scanning of the existing literature. This exercise assisted in the development of the research question to guide the scope of the review. The broad research question this scoping review will attempt to answer: “What is the effect of community awareness interventions on lung cancer in resource-poor settings?” The review will give an overview of the various interventions available, appraise their effectiveness and identify barriers and facilitators to their effectiveness.

### Framework stage 2: Identifying relevant studies

Identification of studies relevant to this review will be achieved by searching electronic databases of the published literature (including published systematic reviews). These electronic databases include Cochrane Library, PubMed, Scopus, MEDLINE, the Cumulative Index to Nursing and Allied Health Literature (CINAHL), PsycInfo, Web of Science, Google Scholar, EMBASE and the Educational Resources Information Centre (ERIC). All reference lists of included studies will be searched to identify additional relevant studies. A variety of grey literature sources will also be searched to ensure that all relevant information is captured. Relevant grey literature databases (such as Grey Literature Report, OpenGrey, Web of Science Conference Proceedings) will be searched to identify studies, reports and conference abstracts applicable to this review (Additional file 2).

The inclusion criteria will include (i) language of publication: English, (ii) timeframe: no time frame, (iii) study setting: resource-poor setting, and (iv) types of articles: randomised controlled trials, intervention studies (including quasi-experimental designs), reports, literature reviews and evidence maps. Studies will be excluded if they do not meet any of the listed criteria.

Search terms will be determined with input from the research team and in consultation with and academic librarian. However, the following are some of the keywords that are likely to be used to search for articles: lung cancer, awareness, community, interventions, and effective. Terms will be searched as both keywords in the title, abstract and/or subject headings (i.e. MeSH terms). The review articles retrieved were then screened for their titles, abstracts and index terms. The articles retrieved from each database will be imported into a reference management software, namely EndNote. Endnote will be used for managing records, keeping track of articles and producing a list of references included in the final review report.

### Framework stage 3: Study selection

The review of articles will have two levels of screening. The first level will be title and abstract review, where two team members will independently screen the title and abstract of all retrieved citations for inclusion against a set of minimum inclusion criteria; the second, full-text review. Initially, a sample of the retrieved articles will be independently screened by two team members to ensure consistent application of the eligibility criteria for inclusion in the review. Any articles deemed relevant by one or both of the reviewers will be included in the full-text review. Inter-rater reliability will be assessed using percentage agreement. Once the percentage agreement reaches 80% or above, advancement to the next stage can be done. For lower agreement, the inclusion and exclusion criteria will be clarified. The team will ensure that the duplicates from the different search databases are eliminated. A member of the team will then screen titles and abstracts of the articles to exclude those that do not meet the eligibility criteria identified in the second stage of the protocol. The full-text articles will be retrieved for the titles and/or abstracts meeting the inclusion criteria.

Disagreements about study eligibility of the sampled articles will be discussed between the two reviewers until a consensus is reached or by arbitration of a third reviewer, if required. The Preferred Reporting Items for Systematic Reviews and Meta-Analyses (PRISMA) flow chart [24–26] will be used to report on the selection process of studies included in this review (see Additional file 1).

### Framework stage 4: Charting the data

Key pieces of information from the abstracts of the selected articles will be collected and sorted. Standard data items will be extracted from the published research literature that will include author, year of publication, study objectives and additional information (i.e. type of study and objectives of the study, intervention strategies on lung cancer awareness and target populations) and will be reported. Based on the preliminary scoping phase, a data extraction framework was developed which will guide the full review articles retrieved from the literature fulfilling the eligibility criteria for inclusion (Table 1). For each article, information on the interventions covered by the review, characteristics of the study populations, setting, length and intensity of the interventions, types of outcomes assessed, information on the effectiveness of the interventions and facilitators and barriers for the implementation of the interventions will be tabled.

**Table 1:** Data extraction framework

Main category	Sub-category	Description
1. Authors		
2. Title		
3. Journal		
4. Year of publication		
5. Objectives of the article		Description of the study objectives
6. Type of study	Primary (knowledge) Multiple outcomes (health-seeking behaviour, knowledge)	RCT, quasi-experimental, etc.
7. Country(ies) where the study was conducted		Where was this study conducted (Geographical distribution)
8. Language		Language used in the intervention/publication
9. Target population	Individual Community, etc.	Individual or group (community) Specific age group Specific sex/gender
10. Setting of the intervention		Community-based, facility-based, urban, rural, resource-poor, etc.
11. Effectiveness		Intended intervention outcomes, results presented by authors, future research directions identified by authors

The framework will be pilot tested by two team members on a sample (30%) of the included studies in order to ensure that the coding framework is consistently applied. Additional categories may emerge during the pilot testing process. If necessary, the categories will be modified and the data extraction framework revised accordingly. Questions arising when piloting the framework will be discussed by the team and possible disagreement will be resolved through team consultations. Missing data may be found on some eligible abstracts, and this will be resolved and documented in consultation with the team members. The authors of those articles will be contacted in an attempt to obtain the required details. If there is no response from the authors in 2 to 3 weeks, this will be considered as no information available. However, the study will be included in the narrative with a mention that an attempt was made to contact the authors for the missing data.

The team members that were piloting the framework will be responsible for independently charting the data from each included review study. Similarly, disagreements in extracted data between the two members will be discussed by these members until a consensus is reached or by arbitration of a third reviewer, if required.

### Framework stage 5: Collating, summarising and reporting the results

This review seeks to compile a summary of findings and then propose a “best” model/intervention in a resource-poor setting broadly present an overview of a particular area of interest. The study will present a narrative account of existing literature using thematic construction based on the different identified intervention types from the selected studies. The analysis of the data collected using the data extraction framework, in this scoping review, will provide information on the body of research that has been conducted on interventions to raise awareness on lung cancer in resource-poor settings. It will be able to provide information on the types of evidence available, the aggregated findings, and offer an outline of the published research rather than an assessment of the quality of individual studies. It would also highlight the effectiveness of the interventions reviewed and their material impact on the target populations. It is also beneficial in identifying gaps in the existing body of knowledge and future research topics.

## Discussion

The results from this scoping review will inform the development of a tailored community lung cancer awareness intervention targeted at selected communities in KwaZulu-Natal, South Africa. This study does not require ethical approval, since the scoping review methodology comprises of reviewing and collecting data from publicly available publications and materials. The results of this scoping review will be disseminated through submitting an article for publication to a scientific journal, conferences, presentations and reports to policymakers and future workshops with professionals involved in lung cancer awareness campaigns. The results will provide a complete synopsis of the interventions on lung cancer awareness and highlight areas where evidence is controversial or missing.

## Additional files

Additional file 1: Proposed PRISMA flowchart. (DOCX 29 kb)

Additional file 2: Draft search strategy and results from three databases. (DOCX 15 kb)

## Data Availability

Not applicable.

## Abbreviations

LIMCs: Low- and middle-income countries
MesH: Medical Subject heading
PRISMA: Preferred Reporting Items for Systematic Reviews and Meta-Analyses
RCT: Randomised controlled trial
SSA: Sub-Saharan Africa
