# Supplementary material for: Mapping the evidence on interventions to raise awareness on lung cancer in resource poor settings: a scoping review protocol
Source: Syst Rev. 2019 Aug 24;8:217. doi: 10.1186/s13643-019-1138-x (PMC6708127; doi:10.1186/s13643-019-1138-x)
Supplement: Supplementary file 1 — Proposed PRISMA flowchart. (DOCX 29 kb) [file 13643_2019_1138_MOESM1_ESM.docx]

Additional file 1

## Identification

## Screening

Records identified through database searching
(n = )

Additional records identified through other sources
(n = )

## Inclusion

Records after duplicates removed
(n = )

Records screened
(n = )

Records excluded
(n = )

Full-text articles assessed for eligibility
(n = )

Full-text articles excluded, with reasons
(n = )

Studies included for data extraction
(n = )

## Eligibility
