# Supplementary material for: Mapping the evidence on interventions to raise awareness on lung cancer in resource poor settings: a scoping review protocol
Source: Syst Rev. 2019 Aug 24;8:217. doi: 10.1186/s13643-019-1138-x (PMC6708127; doi:10.1186/s13643-019-1138-x)
Supplement: Supplementary file 2 — Draft search strategy and results from three databases. (DOCX 15 kb) [file 13643_2019_1138_MOESM2_ESM.docx]

Additional File 2:

Table 1: Draft search strategy and results from three databases

| **Date of search** | **Search engine used** | **Keyword search** | **Number of publications retrieved** |
| --- | --- | --- | --- |
| 16/05/2019 | Google Scholar | lung cancer, awareness, "community interventions", and effective | 1680 |
| 16/05/2019 | PubMed | lung cancer, awareness, community interventions | 17 |
| 16/05/2019 | Web of Science | “lung cancer”[Title/Keywords/Abstract] AND “awareness”[Title/Keywords/Abstract] AND “interventions”[Title/Keywords/Abstract] | 173 |
